# In situ prepared tungsten(VI) oxide supported Pd0 NPs, remarkable activity and reusability in H2 releasing from dimethylamine borane

**DOI:** 10.55730/1300-0527.3315

**Published:** 2021-11-25

**Authors:** Seda KARABOĞA

**Affiliations:** Department of Chemistry, Faculty of Arts and Science, Bolu Abant İzzet Baysal University, Bolu, Turkey

**Keywords:** Pd^0^ Nanoparticles, tungsten(VI) Oxide, dehydrogenation, dimethylamine Borane, heterogeneous Catalyst

## Abstract

The study reports preparation, characterization, and catalytic activity of Pd^0^/WO_3_ nanoparticles in H_2_ evolution from dimethylamine borane (DMAB). The active catalyst called Pd^0^/WO_3_ NPs were obtained from the in situ reduction of Pd^2+^/WO_3_ precatalyst and tested in H_2_ releasing from DMAB. Pd^0^/WO_3_ NPs were found as remarkable catalysts providing a turnover frequency value of 948.0 h^−1^ in H_2_ releasing from DMAB at 60.0 ± 0.5 °C. The results of some advanced analytical techniques reveal that Pd^0^ NPs show uniform dispersion on the surface of tungsten(VI) oxide (WO_3_) with 5.85 ± 0.57 nm of particle size. The reducible nature of tungsten(VI) oxide improves the catalytic efficiency of Pd^0^/WO_3_ NPs in H_2_ generation from DMAB. Pd^0^/WO_3_ NPs were found as active catalysts even after the 6th run of dehydrogenation reaction. The report also covers the kinetic performance of the catalyst.

## 1. Introduction

In the future of sustainable energy, the usage of hydrogen as an energy carrier to store energy appears inevitable. Hydrogen being a clean, sustainable, and efficient energy carrier is one of the appropriate candidates for the storage of energy [1]. It is stored energy as chemically, transport energy and used as a fuel [2]. The hydrogen based energy system called hydrogen economy is an advanced technology system [3]. It is possible to provide sustainable energy using this policy without polluting the environment. However, the safe storage of hydrogen in systems where it will be used as an energy carrier is important and to overcome obstacles in this regard. In recent years, boron compounds are suitable candidates for solid hydrogen storage materials such as ammonia borane, hydrazine borane, and dimethylamine borane [4, 5, 6]. Among the solid hydrogen storage materials, dimethylamine borane (DMAB, (CH_3_)_2_NH.BH_3_) is a prominent B-N compound [7]. In the presence of a suitable catalyst, one equivalent of hydrogen could be released from dimethylamine borane [5, 8, 9] (Equation 1).


(1)

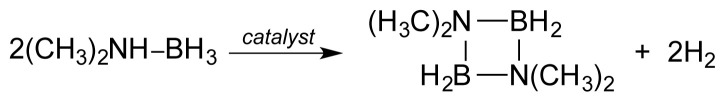


The rate of hydrogen evolution depends on metal catalysts used in dehydrogenation reactions [10, 11]. Among transition metal nanoparticles, especially ruthenium has been used as a catalyst in dehydrogenation reactions [10, 12, 13, 14, 15]. However, there are few reports on the usage of palladium based on heterogeneous catalyst in H2 evolution from DMAB: three of them are monometallic [16, 17, 18] and five of them are bimetallic [19, 20, 21, 22] or trimetallic [23] that act as heterogeneous catalysts. The main problem of the metal nanoparticles acting as the active catalyst is that they are not stable as thermodynamically during the reaction and they tend to be agglomerate. So, they need supporting materials to prevent the agglomeration of the metal nanoparticles. WO3 being one of the suitable supporting materials for transition metal nanoparticles has been used in various reactions such as oxidation of methanol and ethanol [24, 25], dehydrogenation of 2-butanol [26], hydrolysis of ammonia borane [27], decomposition of formic acid [28]. Herein, WO3 was used as a supporting material for palladium(0) nanoparticles due to its reducible nature. WO3 can readily go to reduction under reaction conditions and this situation leads to the excess negative charge on the surface which enhances the catalytic performance of the catalyst depending on strong interaction between metal and support [28]. This unique property of WO3 gives a special chance that it could be used several types of reactions. Palladium(II) acetylacetonate was used as a precursor and impregnated on the surface of the WO3. The precatalyst, Pd2+/WO3 was trialed in dehydrogenation of dimethylamine borane. The reduction of Pd2+ ions on the surface of WO3 leads to Pd0/WO3 which act as an active catalyst during the reaction course. Pd0/WO3 NPs are found as active catalysts providing a turnover frequency value of 948.0 h-1 in H2 evolution from DMAB at 60.0 ± 0.5 °C. The reusability tests showed that Pd0/WO3 NPs are tremendously stable catalysts even after the 6th run of dehydrogenation reaction. The Pd0/WO3 catalyst was collected from the reaction medium and characterized by XRD, TEM, XPS techniques. The study also covers the kinetic performance of the catalyst.

## 2. Experimental

### 2.1. Materials

Palladium(II) 2,4-pentanedionate (Pd(C_5_H_7_O_2_)_2_), toluene, and dimethylamine borane ((CH_3_)_2_NH.BH_3_, 97%) were purchased from Aldrich. Tungsten(VI) oxide (WO_3_, 99.9%) was bought from Nanografi. The glassware used in dehydrogenation reactions was cleaned with acetone and dried in an oven for 12 h.

### 2.2. Instrumentation

The instrumentation part is the same as the one given in our previous work, except for TEM analysis [11]. TEM images were obtained by Hitachi HT-7700 operating at 120kV.

### 2.3. In situ preparation of Pd^0^/WO_3_ NPs in H_2_ generation reactions

Pd^0^/WO_3_ NPs were obtained from the in situ reduction of Pd^2+^ ions on the surface of support during the catalytic reaction. In this method, Pd^2+^ ions and supporting material, WO_3_ were mixed for 1 h to adherence to Pd^2+^ ions. A 2.91 mM stock solution of palladium(II) acetylacetonate was prepared by dissolving 8.85 mg Pd(II) acetylacetonate in 10.0 mL of toluene. The defined amount of the stock solution was transposed to the reactor including 100.0 mg of WO_3_ and stirred at 25.0 ± 0.5 °C for 1 h. The temperature of the mixture was fixed at 60 °C by circulating the water around the reaction tube. Then, DMAB was added to the reactor and hydrogen evolution was followed by measuring the water level inside the glass tube.

### 2.4. Determination of catalytic performance of Pd^0^/WO_3_ NPs with different palladium loadings

In order to determine the optimum Pd loading in the catalyst sample, a series of experiments were performed using 100 mg WO_3_ with different Pd loadings (0.5, 1.0, 1.5, and 2.0 % wt. Pd) in 10 mL of a toluene solution. 100 mM DMAB (60.10 mg) was used for each experiment. The highest turnover frequency value was obtained for 1.0% Pd wt. and found as 948.0 h^−1^ at 60.0 ± 0.5 °C in H_2_ generation reaction. Therefore, Pd^0^/WO_3_ NPs with 1.0% Pd loading were used for further dehydrogenation experiments.

### 2.5. Leaching test of Pd^0^/WO_3_ NPs catalyst in H_2_ generation reaction

After the first run of reaction, the reaction tube was settled down to the collapse of the catalyst. Then, the liquid part of the solution was filtered by using a feeding tube and collected. The filtrate part and solid part of the sample were separately tested in hydrogen releasing from DMAB. While the liquid part of the sample does not indicate any catalytic activity, the solid part is still active in the reaction.

### 2.6. Recyclability test for Pd^0^/WO_3_ in H_2_ generation reaction

The catalytic activity of Pd^0^/WO_3_ NPs was tested in subsequent runs of dehydrogenation reaction. After the first run of the reaction, the same amount of DMAB was added to the reaction solution and the reaction was followed. The same experimental procedure was repeated for the subsequent runs.

## 3. Results and discussion

### 3.1. Characterization of Pd^0^/WO_3_ NPs

**Figure 1:**
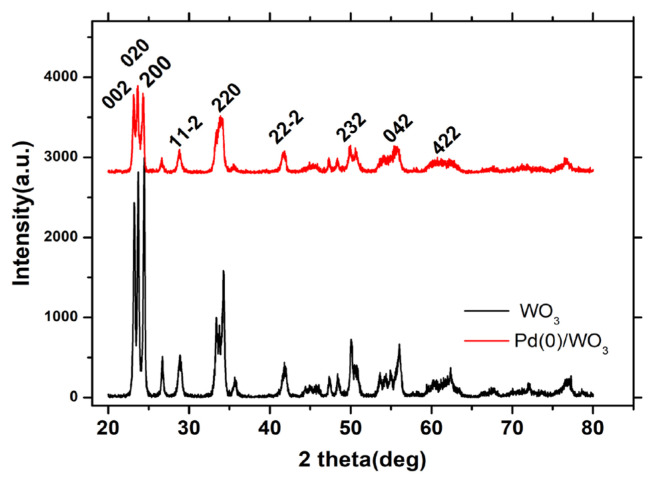
XRD pattern of bare WO_3_ and 1.0% Pd wt. loaded WO_3_ samples.

After determination of catalytic efficiency of Pd^0^/WO_3_ NPs, they were characterized by analytical techniques such as XRD, TEM, and XPS. Figure 1 shows the XRD patterns of bare WO_3_ and WO_3_ supported Pd^0^ NPs after the catalytic dehydrogenation reaction of DMAB. No significant change has been seen in WO_3_ crystallinity after Pd loading on the surface of the support. There is no additional peak that belongs to metallic Pd in XRD pattern in Figure 1 due to the low Pd loading in the sample. (PDF Card No: 01-083-0950).

**Figure 2:**
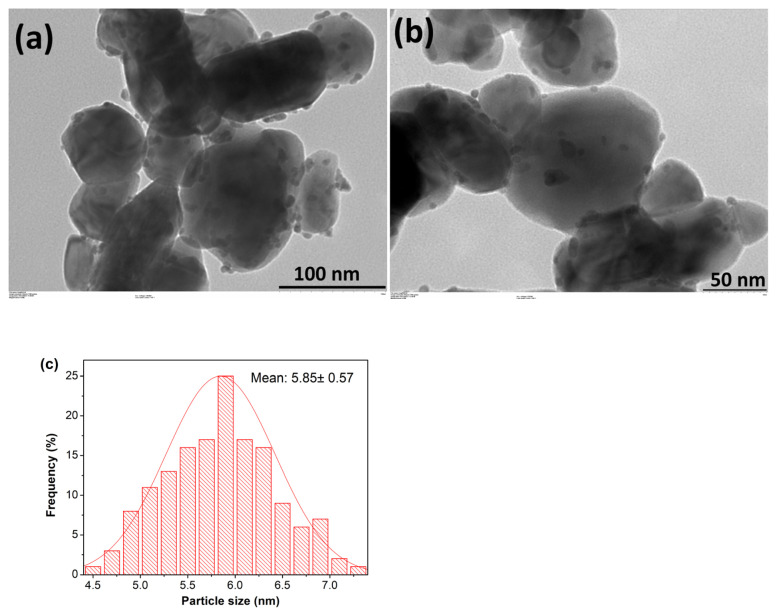
a, b) TEM images of WO_3_ supported Pd^0^ NPs with 1.0% Pd wt. c) Particle size diagram of Pd^0^ NPs on the surface of WO_3_.

TEM images of Pd^0^/WO_3_ NPs given in Figure 2 depict that Pd^0^ NPs are well dispersed on the surface of WO_3_. The mean particle size of Pd^0^ NPs supported on the surface of WO_3_ was determined from the TEM images by measuring more than 150 nontouching particles on the images. The histogram in Figure 2c shows the particle size distribution of Pd^0^ NPs. WO_3_ supported Pd^0^ NPs are well dispersed on the support with an average particle size of 5.85 ± 0.57 nm.

**Figure 3:**
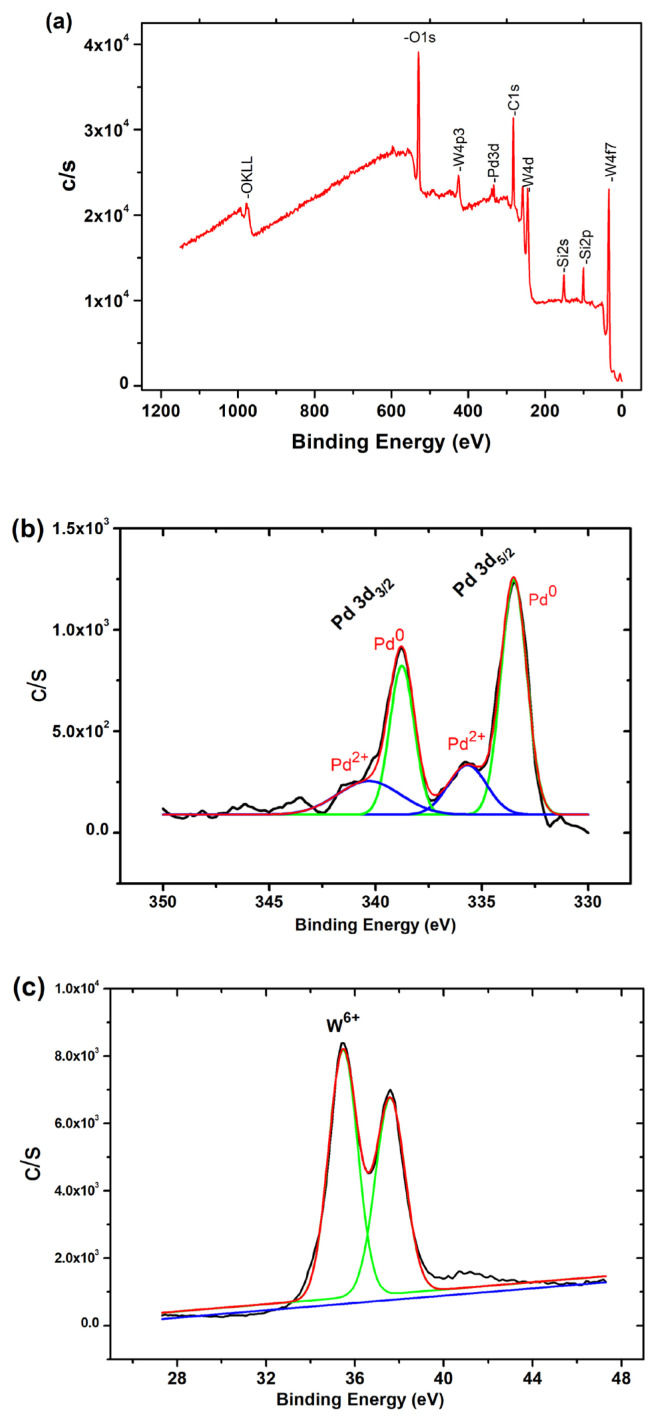
XPS spectra of Pd^0^/WO_3_ sample. a) Full range XPS spectra, b) Deconvolution of Pd 3d range, c) XPS spectrum of W4f_7/2_ bands.

The survey scan XPS analysis and high resolution spectra of Pd^0^/WO_3_ sample could be seen in Figure 3. The survey scan spectrum of Pd^0^/WO_3_ NPs shows that Pd, W, O and C existed in the sample (Figure 3a). For Pd, deconvolution of Pd 3d photoelectrons was obtained as seen in Figure 3b. The high resolution spectra of Pd 3d scan of Pd^0^/WO_3_ sample indicates two main peaks at 343.8 and 340.0 eV which could be attributable to 3d_5/2_ and 3d_3/2_ of metallic palladium, respectively [29, 30]. The peaks at higher binding energy may be attributed to Pd being in a 2+ oxidation state [31, 32]. The two peaks with low intensity have resulted from the oxidation of palladium metal in Pd^0^/WO_3_ sample during the XPS analysis. The color of WO_3_ turns dark blue during the reaction course, which indicates the reduction of W^6+^ to W^5+^ with the addition of DMAB. However, well fitted W4f core-level XPS spectrum in Figure 3c indicates that no noticeable peak for W^5+^ state due to the oxidation of sample subjugated to air during the sampling.

### 3.2. Catalytic performance of Pd^0^/WO_3_ NPs

**Figure 4:**
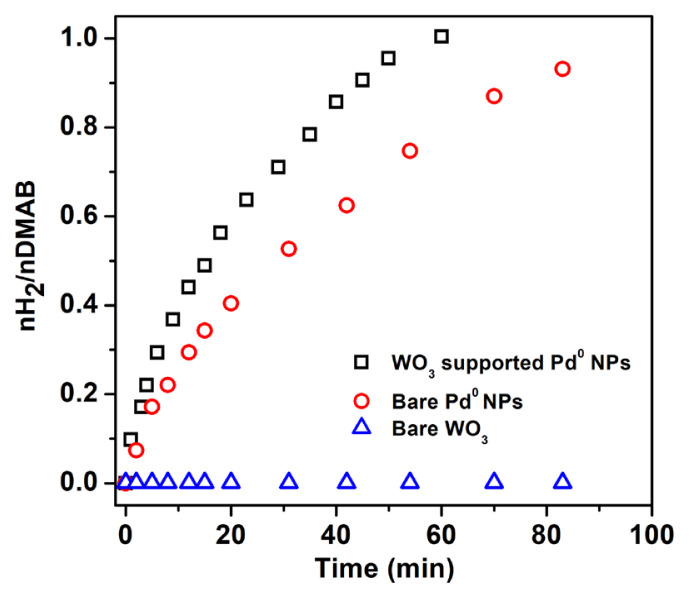
Comparison of hydrogen evolution from DMAB in the presence of bare Pd^0^ NPs and WO_3_ supported Pd^0^ NPs.

**Figure 5:**
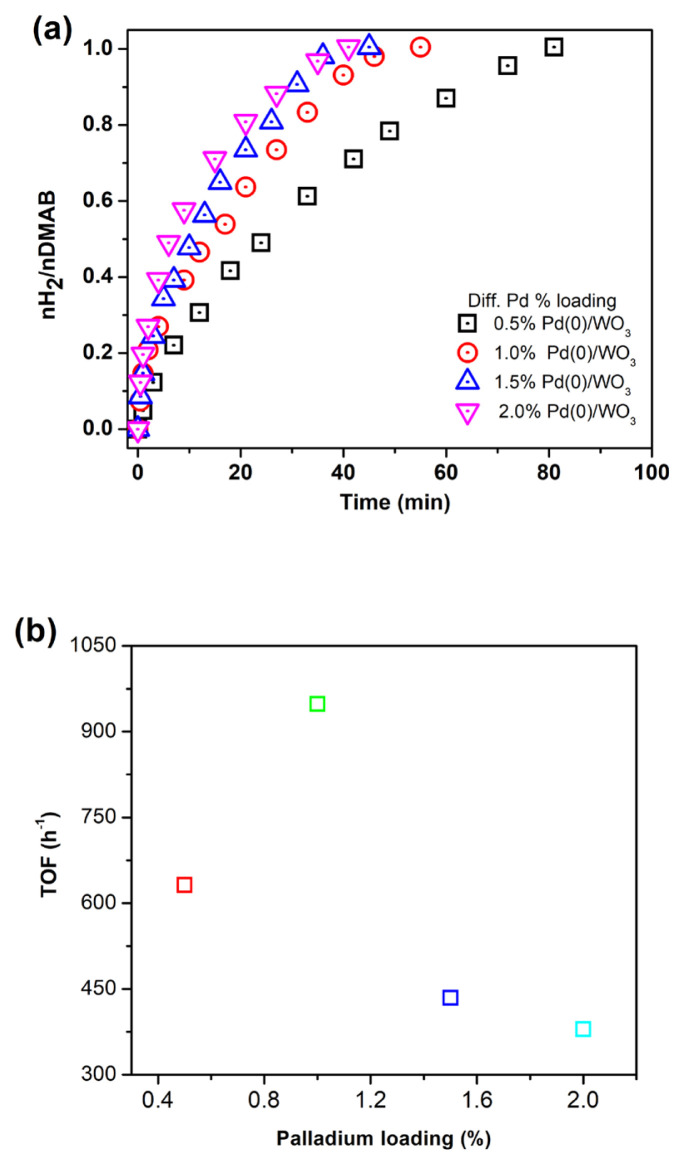
a) H_2_ evolution graph with different Pd loadings (0.5, 1.0, 1.5, and 2.0 % wt.) b) TOF vs Pd loading in the catalyst.

Hydrogen evolution from DMAB was investigated in the presence of Pd^0^/WO_3_ NPs catalyst. Before testing the Pd^0^/WO_3_ NPs, bare Pd^0^ NPs were tested in hydrogen releasing from DMAB at 60.0 ± 0.5 °C. The comparison of bare Pd^0^ NPs and WO_3_ supported Pd^0^ NPs given in Figure 4 shows that WO_3_ enhances the catalytic activity of Pd^0^ NPs owing to prevent the agglomeration of them during the reaction course. The catalytic performance of Pd^0^/WO_3_ NPs changes depending on palladium loadings in the sample. Figure 5a indicates the hydrogen evolution varying with palladium loadings of the Pd^0^/WO_3_ sample at 60.0 ± 0.5 °C. Hydrogen evolution rates and initial TOF values vary depending on palladium loading in the catalyst. The highest turnover frequency was found as 948 h^−1^ for 1.0% Pd wt loading of Pd^0^/WO_3_ sample. Figure 5b depicts palladium loading versus the TOF value of the catalyst. The volcano shape of the graph describes the relationship needed to be optimized between the palladium and WO_3_ support. The TOF value reaches a maximum and then decreases with the increment of palladium loading which is related to the supporting capacity of WO_3_ support and agglomeration of Pd^0^ NPs on the surface of the support. This type of relation has been previously reported for copper and palladium catalyst in hydrogen generation reactions [11, 16]. Therefore, Pd^0^/WO_3_ NPs with 1.0% wt Pd were used for further experiments.

**Figure 6:**
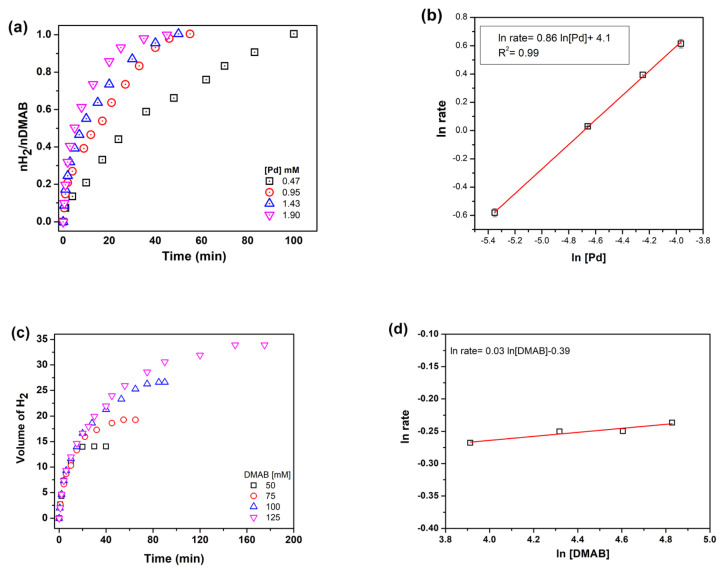
a) H2 evolution graph with different Pd concentrations b) ln rate vs. ln Pd c) Hydrogen evolution graph with different DMAB concentrations d) ln rate vs. ln DMAB.

Dehydrogenation reactions were performed with different catalyst concentrations to determine the reaction order with respect to catalyst. Figure 6a shows hydrogen evolution from DMAB in the presence of Pd^0^/WO_3_ NPs with different palladium concentrations. Hydrogen evolution rate was determined by the linear part of each line and the slope of the line was found as 0.86 indicating that dehydrogenation of DMAB is approximately first order with respect to palladium catalyst concentration (Figure 6b).

The effect of substrate concentration was also determined by performing a set of experiment while keeping the metal concentration constant at 0.95 mM. It can be seen that from the slope of the line in Figure 6c, the hydrogen generation rate from the catalytic dehydrogenation of DMAB is actually independent of DMAB concentration. So, the reaction catalyzed by Pd(0)/WO_3_ NPs is zero order with respect to the substrate concentration (Figure 6d). Thus, the rate law of dehydrogenation reaction can be given as Eq 2:


$$\text{Rate}={\text{k}}^{\text{app}}[\text{Pd}]$$

**Figure 7:**
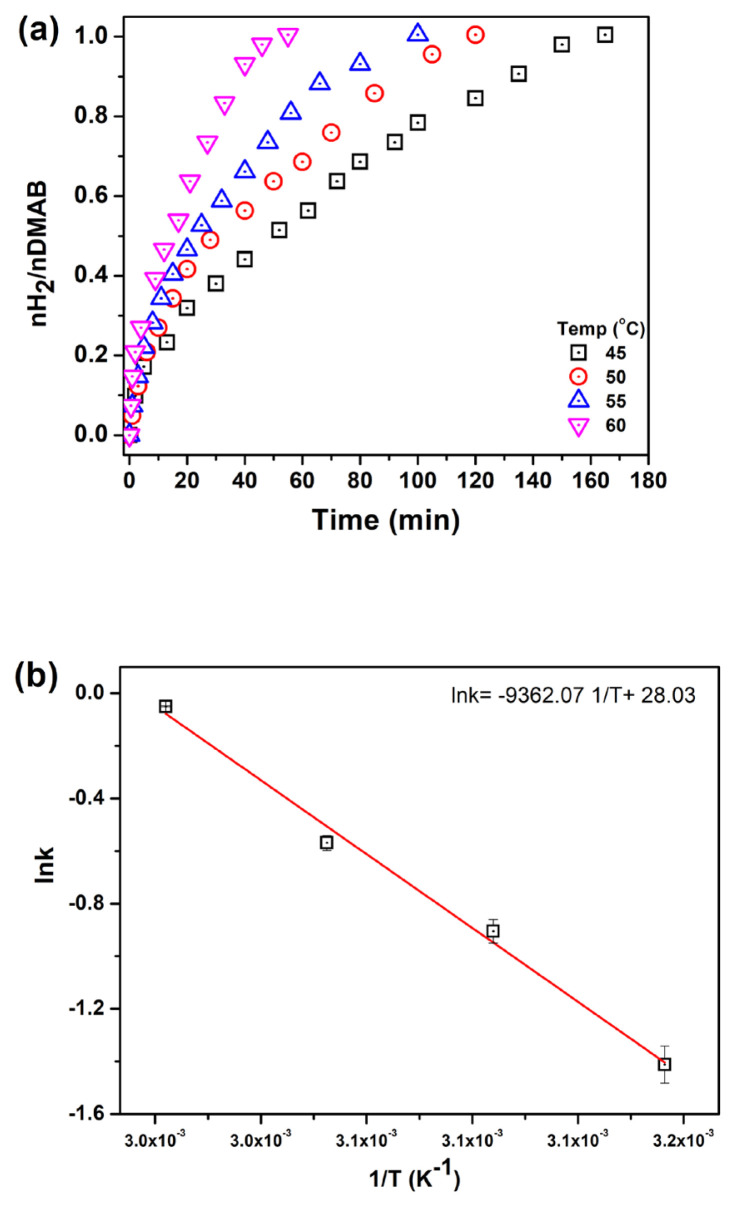
a) H_2_ evolution from DMAB at different temperatures, b) Arrhenius plot.

H_2_ production reactions were studied at various temperatures (Figure 7a). The activation energy of the catalytic dehydrogenation of DMAB was determined from the slope of Arrhenius plot [33] to be Ea = 77.84 kJ/mol which is comparable to the previously reported values [18, 20, 21].

**Figure 8:**
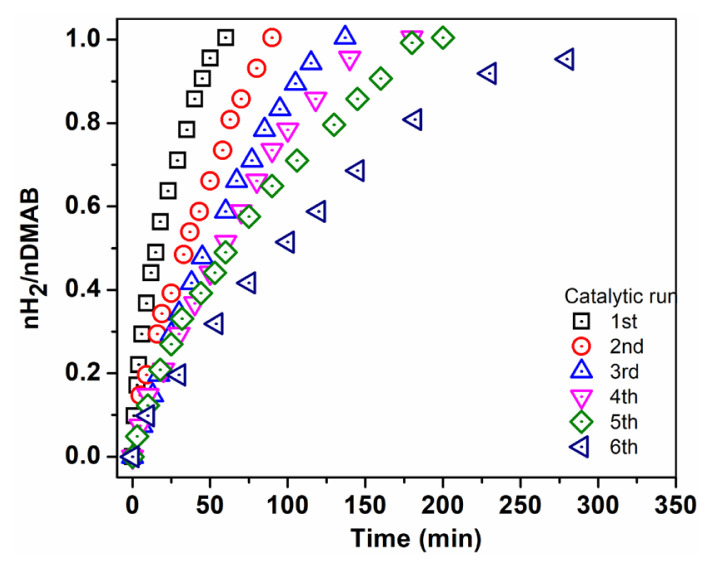
Catalytic performance of Pd^0^/WO_3_ in subsequent run of dehydrogenation.

Recyclability of the catalyst is very important as well as catalytic activity in terms of practical applications. Recyclability of Pd^0^/WO_3_ NPs was performed in the dehydrogenation of DMAB at 60.0 ± 0.5 °C and it is seen in Figure 8 that Pd^0^/WO_3_ NPs are still impressive catalysts after the 6th run of the reaction. The special property of WO_3_ support undergoing reduction in suitable conditions plays a significant role in the catalytic usage of Pd^0^ NPs in H_2_ evolution reactions. The reducibility feature of WO_3_ leads to a partial reduction of W6+ to W5+ and excessive charge on the surface of support which promotes the strong interaction between metal and support.

**Figure 9:**
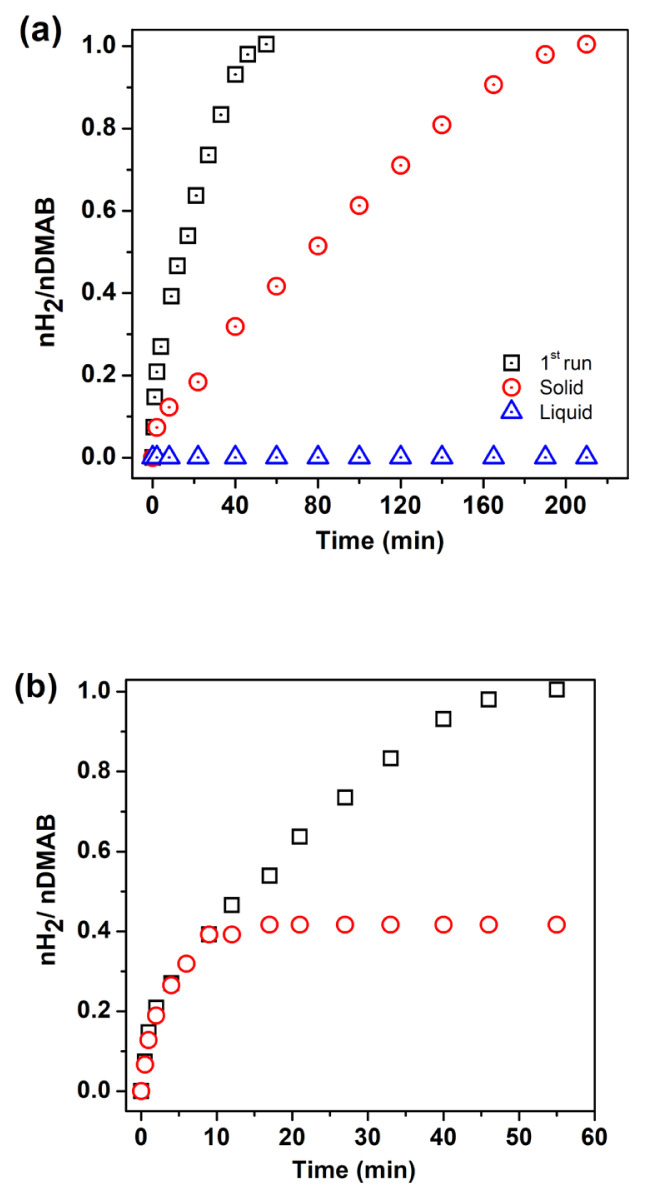
a) Leaching, b) Poisoning test for Pd^0^/WO_3_ NPs.

Leaching experiment and poisoning experiments were also studied for Pd^0^ NPs supported on the surface of WO_3_. After the first run of the catalytic run, the reaction solution was filtered by inverse filtration using a feeding tube and micropipette. The solid and liquid part of the solution was tested separately in H_2_ evolution reaction from DMAB. While the liquid part of the solution does not show any catalytic activity, the solid part retained its catalytic activity in the dehydrogenation reaction (Figure 9a). For the poisoning test, a typical experiment was started in the presence of 0.95 mM Pd catalyst at 60.0 ± 0.5 °C. After the liberation of 0.4 equiv H_2_ from the dehydrogenation reaction, 0.2 equivalent of CS_2_ was injected into the reaction medium. The reaction stopped immediately after the addition of CS_2_ due to the inhibition of the catalyst (Figure 9b). The result of leaching and poisoning tests reveal that catalyst is kinetically competent and dehydrogenation of DMAB is heterogeneous catalysis.

## 4. Conclusion

WO_3_ supported Pd^0^ NPs were obtained from the reduction of Pd^2+^ ions impregnated on the surface of WO_3_ and tested as a catalyst in H_2_ production from DMAB at 60.0 ± 0.5 °C. The results of the characterization techniques reveal that Pd^0^ NPs are well dispersed on the surface WO_3_ with an average particle size of 3.73 ± 0.58 nm. The initial TOF value reaches a maximum and decreases with the enhancement of Pd loading in the sample. The highest TOF value was obtained as 948.0 h^−1^ for 1.0% Pd wt. in Pd^0^/WO_3_ sample. The strong interaction between the WO_3_ support and Pd NPs provides the high catalytic activity in H_2_ evolution reaction. Especially reducible nature of WO_3_ increases the recyclability of Pd^0^ NPs in dehydrogenation reaction. Under reducing condition, WO_3_ goes to a partial reduction of W6+ to W5+ ions on the surface as proved by observation of dark blue color. The excessive negative electron charge on the surface promotes the metal-support interaction. The facile preparation of Pd^0^/WO_3_ NPs and catalytic performance in dehydrogenation reactions make them a potential candidate to be utilized in H_2_ releasing using DMAB as a solid hydrogen storage material.
